# Supplementary material for: Isolation and Anticancer Progression Evaluation of the Chemical Constituents from Bridelia balansae Tutcher
Source: Molecules. 2023 Aug 21;28(16):6165. doi: 10.3390/molecules28166165 (PMC10457964; doi:10.3390/molecules28166165)
Supplement: Supplementary file 1 [file molecules-28-06165-s001.zip › molecules-2562110-supplementary.pdf]

Support information

Isolation and bioactivity evaluation of the chemical constituents from *Bridelia balansae* Tutcher

Lihan Zhao [1,2,†], Wen-Jian Xie [1,†], Yin-Xiao Du [1,†], Yi-Xuan Xia [1], Kang-Lun Liu [1], Chuen Fai Ku [1], Zihao Ou [4], Ming-Zhong Wang [3,*] and Hong-Jie Zhang [1,*]

1 School of Chinese Medicine, Hong Kong Baptist University, Kowloon, Hong Kong SAR, China

2 Department of Biology, Georgia State University, Atlanta, GA, USA

3 College of Pharmacy, Shenzhen Technology University, Shenzhen, China

4 Department of Materials Science and Engineering, Stanford University, Stanford, CA 94305, USA

* Correspondance : wangmingzhong@sztu.edu.cn (M.-Z.W.) ; zhanghj@hkbu.edu.hk (H.-J.Z.) ;

† These authors contributed equally to this work.

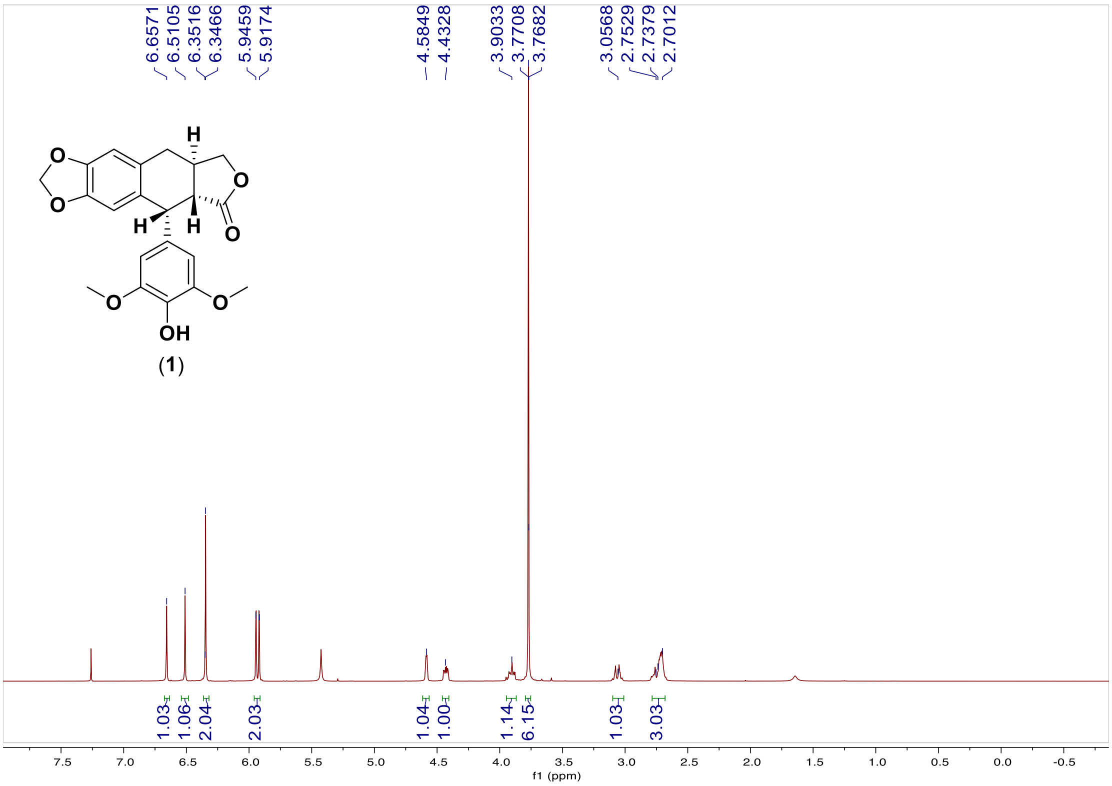

Figure S1. The proton NMR spectrum of compound **1**

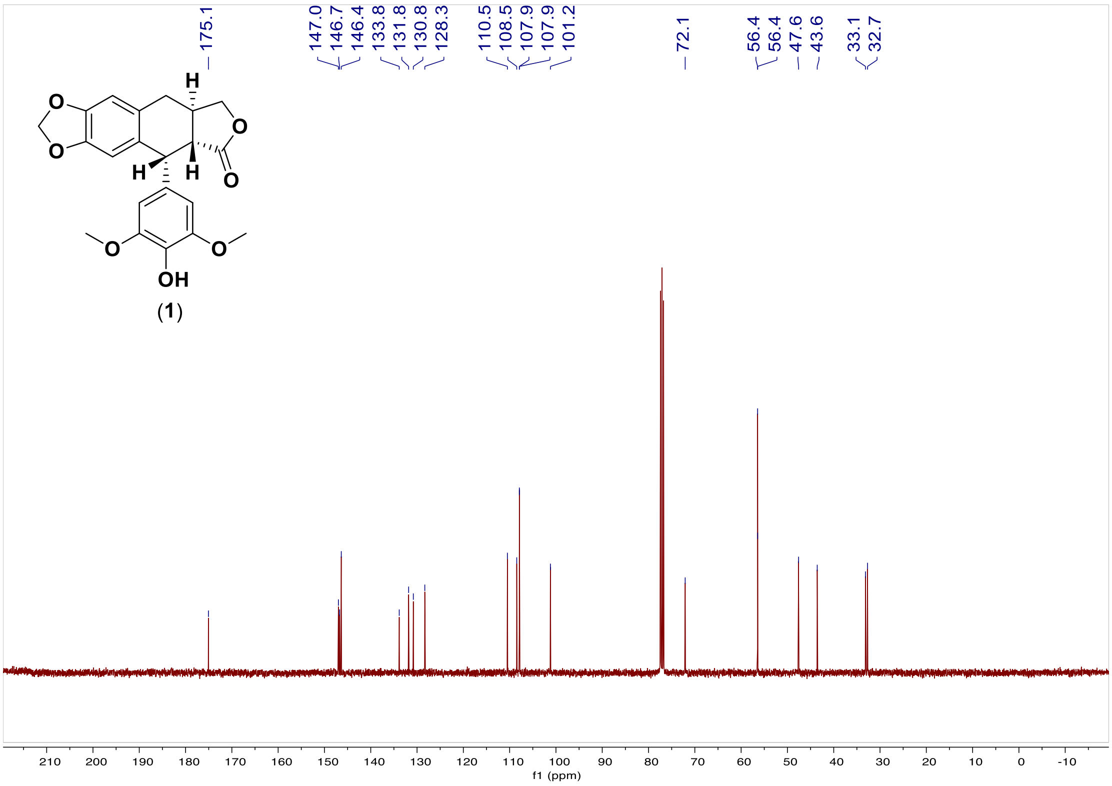

Figure S2. The [13]carbon NMR spectrum of compound **1**

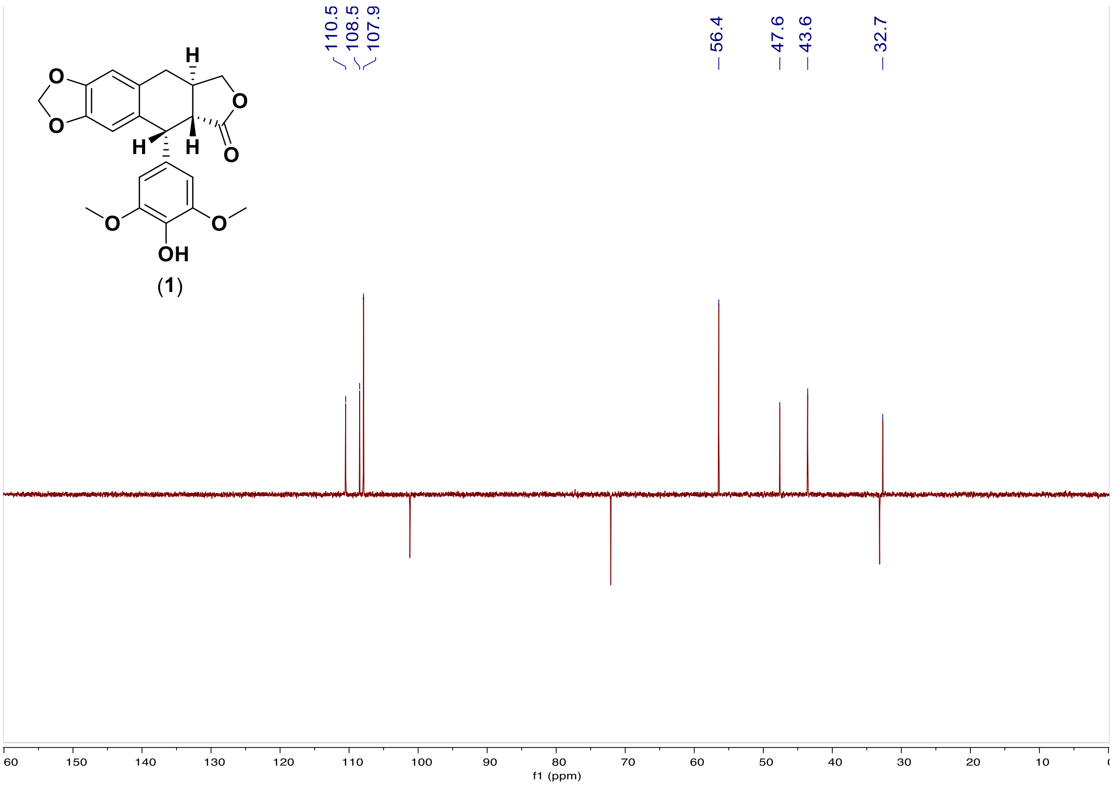

Figure S3. The DEPT135 NMR spectrum of compound **1**

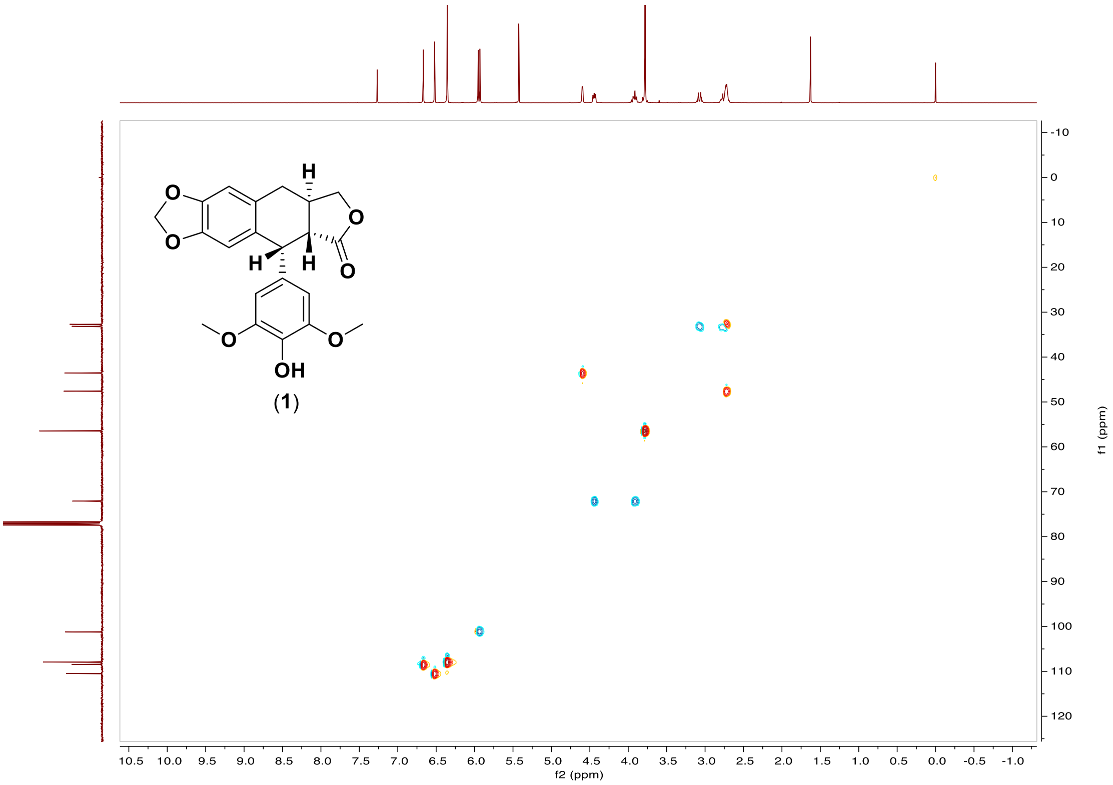

Figure S4. The HSQC NMR spectrum of compound **1**

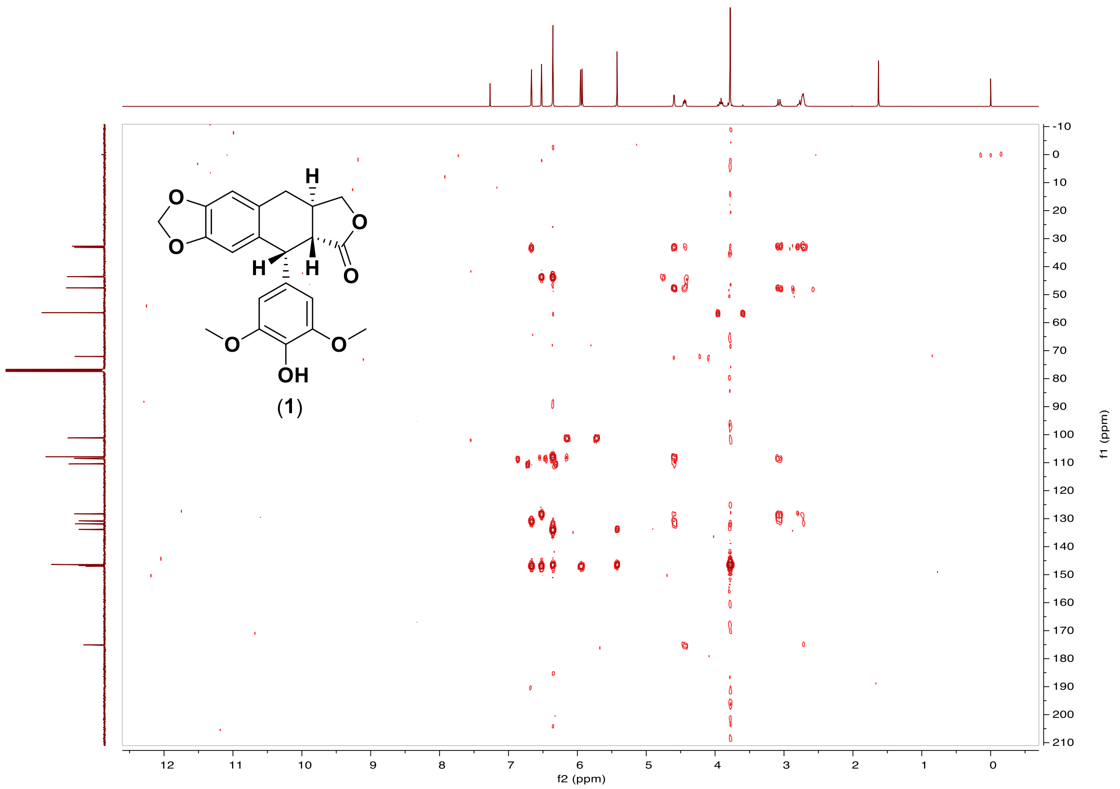

Figure S5. The HMBC NMR spectrum of compound **1**

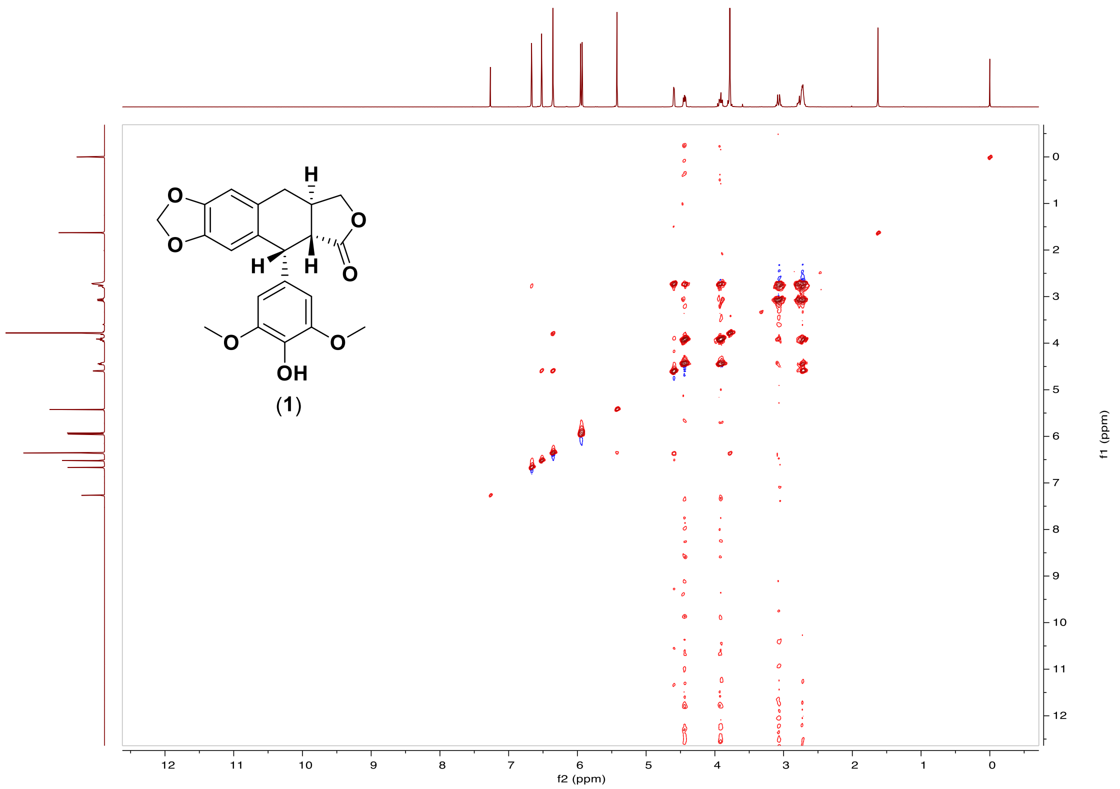

Figure S6. The ^{1}H-^{1}H COSY NMR spectrum of compound **1**

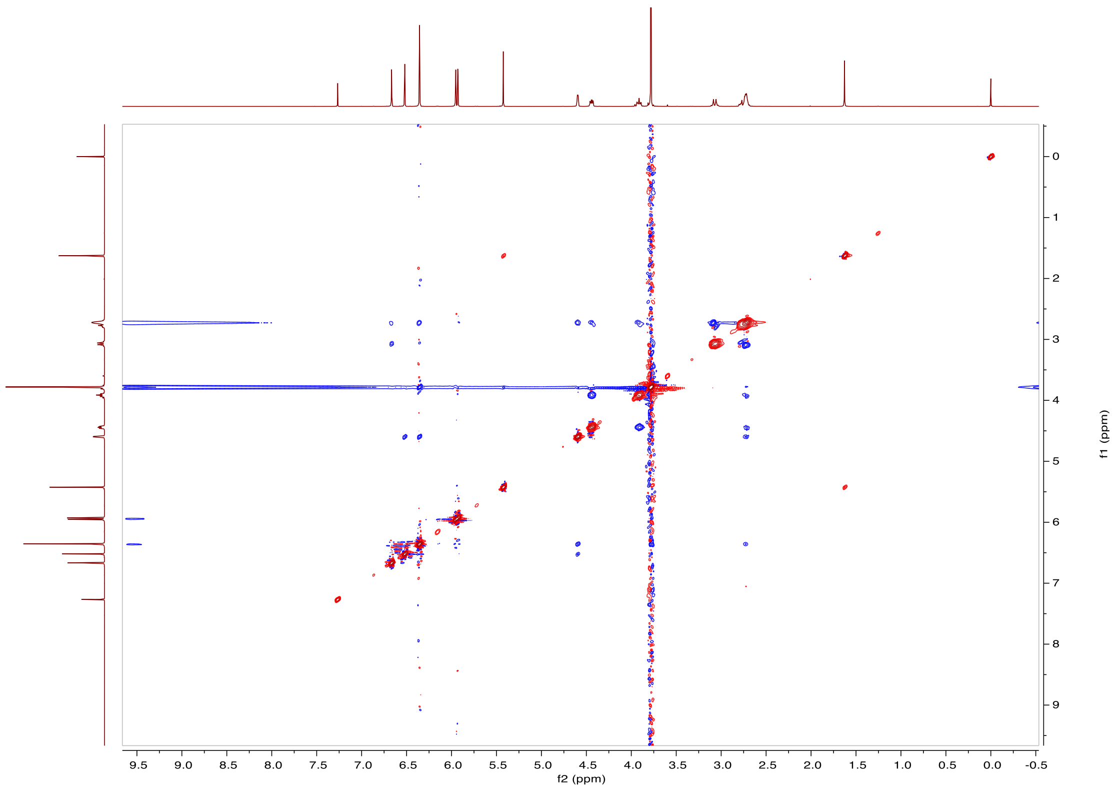

Figure S7. The NOESY NMR spectrum of compound **1**

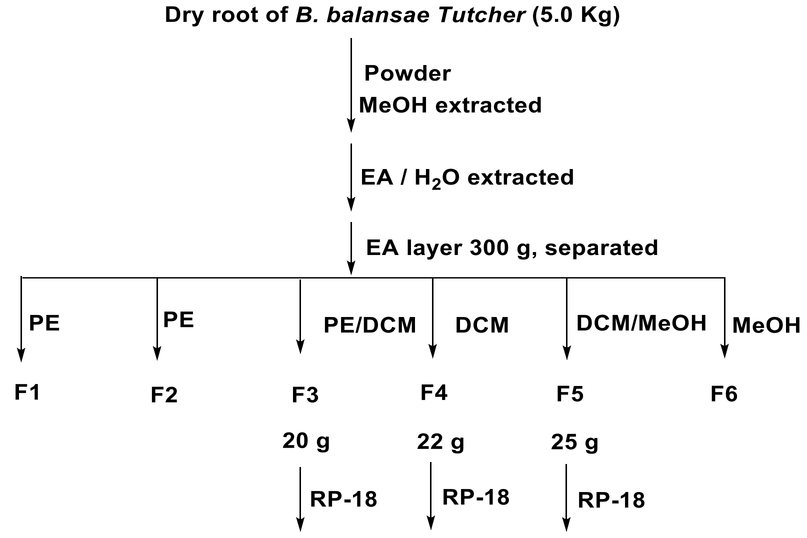

Figure S8. The extraction and fraction flow chart of air-dried root of *B. Balansae*

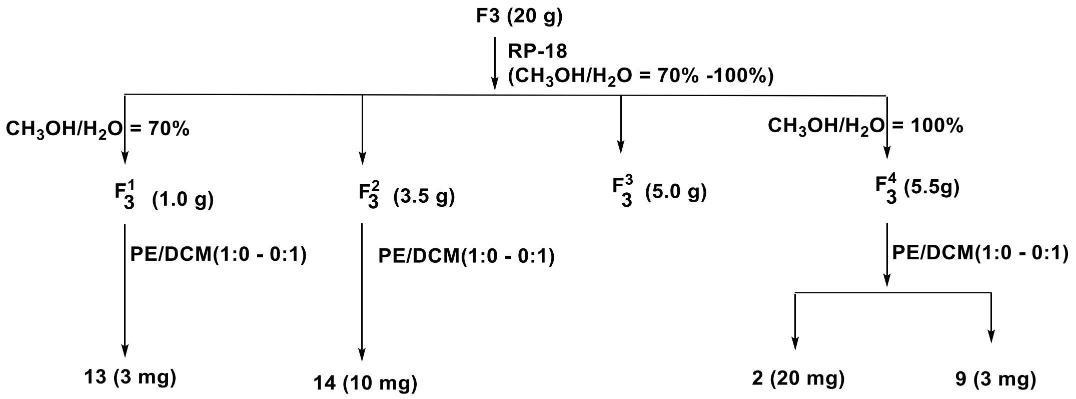

Figure S9. The isolation flow chart of sub fraction F3

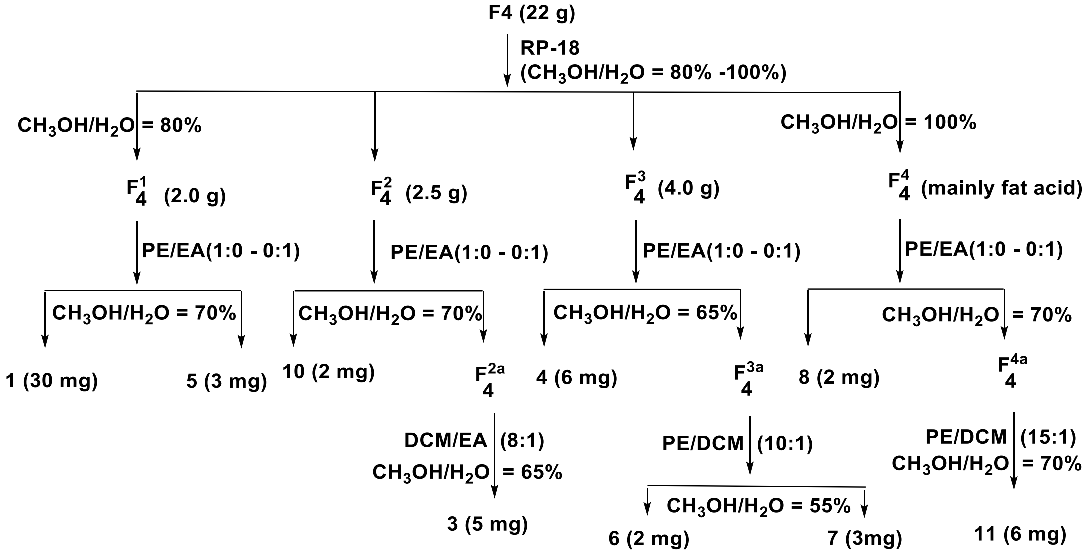

Figure S10. The isolation flow chart of sub fraction F4

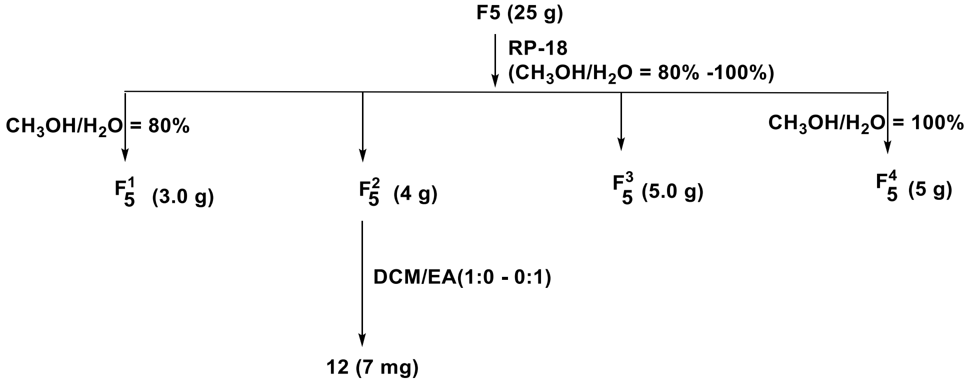

Figure S11. The isolation flow chart of sub fraction F5
